# RS‐1 enhances CRISPR‐mediated targeted knock‐in in bovine embryos

**DOI:** 10.1002/mrd.23341

**Published:** 2020-03-30

**Authors:** I. Lamas‐Toranzo, A. Martínez‐Moro, E. O´Callaghan, G. Millán‐Blanca, J.M. Sánchez, P. Lonergan, P. Bermejo‐Álvarez

**Affiliations:** ^1^ Animal Reproduction Department INIA Madrid Spain; ^2^ Procreatec Madrid Spain; ^3^ School of Agriculture and Food Science University College Dublin Dublin Ireland

**Keywords:** bovine, DNA repair, embryo, gene editing, RS‐1

## Abstract

Targeted knock‐in (KI) can be achieved in embryos by clustered regularly interspaced short palindromic repeats (CRISPR)‐assisted homology directed repair (HDR). However, HDR efficiency is constrained by the competition of nonhomologous end joining. The objective of this study was to explore whether CRISPR‐assisted targeted KI rates can be improved in bovine embryos by exposure to the HDR enhancer RS‐1. In vitro produced zygotes were injected with CRISPR components (300 ng/µl Cas9 messenger RNA and 100 ng/µl single guide RNA against a noncoding region) and a single‐stranded DNA (ssDNA) repair template (100 ng/µl). ssDNA template contained a 6 bp *Xba*I site insert, allowing targeted KI detection by restriction analysis, flanked by 50 bp homology arms. Following microinjection, zygotes were exposed to 0, 3.75, or 7.5 µM RS‐1 for 24 hr. No differences were noted between groups in terms of development or genome edition rates. However, targeted KI rates were doubled in the group exposed to 7.5 µM RS‐1 compared to the others (52.8% vs. 25% and 23.1%, for 7.5, 0, and 3.75 µM, respectively). In conclusion, transient exposure to 7.5 µM RS‐1 enhances targeted KI rates resulting in approximately half of the embryos containing the intended mutation, hence allowing direct KI generation in embryos.

## Graphical abstract

RS‐1 biases DNA repair towards homologous directed repair (HDR). Enhancing HDR increases the efficiency of clustered regularly interspaced short palindromic repeats (CRISPR)‐mediated targeted KI. Following transient RS‐1 exposure half of the injected bovine embryos contain the intended DNA insertion.

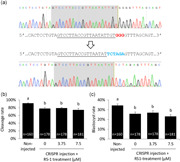

## 1 INTRODUCTION

The eruption of genome editing by site‐specific endonucleases has enabled multiple applications in farm animals aimed at improving animal production, modifying animal products for diverse purposes, or understanding the molecular mechanisms behind biological processes involved in animal or human disease (Lamas‐Toranzo et al., 2017). However, while those applications requiring the ablation of a gene are efficiently achieved by the generation of random insertion‐deletions (indels) following oocyte or zygote microinjection (H. Wang et al., 2013), the precise modification of specific bases is still inefficient (Singh, Schimenti, & Bolcun‐Filas, 2015). These precise DNA modifications, achieved by targeted knock‐in (KI), are required for applications such as allele introgression, that is, the replication of an already existing desirable allele in a target breed/genetic line (Tan et al., 2013), or the expression of a protein of interest under an endogenous promoter (Lillico et al., 2016).

Targeted KI, also known as precise genome editing, relies on homology‐directed repair (HDR), an endogenous DNA repair mechanism of eukaryotic cells. When site‐specific endonucleases such as clustered regularly interspaced short palindromic repeats (CRISPR) induce a DNA double‐stranded break (DSB) at the target locus, the damage is usually repaired by one of two endogenous mechanisms: nonhomologous end joining (NHEJ) or HDR. Both canonical NHEJ or the alternative microhomology‐mediated end‐joining (MMEJ), are error‐prone, often generating indels at the repaired locus. As NHEJ does not use any recombination template, it cannot mediate large insertions. Precise modification of small sequences is also largely unachievable by NHEJ due to its random nature, although considerable efforts have been devoted to predicting indels generated following canonical NHEJ or MMEJ repair as a means of generating specific point mutations (Iyer et al., 2019; Shen et al., 2018). In contrast to NHEJ, HDR uses a template DNA as a guide to repair the DSB. The HDR template can be artificially synthesized to contain the intended DNA modification, which would be introduced at the desired locus, hence allowing targeted KI (Brinster et al., 1989). As DSB stimulates HDR in mammalian cells (Rouet, Smih, & Jasin, 1994), the combination of CRISPR + HDR has boosted the efficiency of targeted KI compared to HDR alone (H. Yang, Wang, & Jaenisch, 2014). However, as NHEJ is the predominant DSB repair pathway in vertebrates (Sonoda, Hochegger, Saberi, Taniguchi, & Takeda, 2006) and the indels generated by NHEJ often prevent target recognition by CRISPR (Lamas‐Toranzo, Ramos‐Ibeas, Pericuesta, & Bermejo‐Alvarez, 2018), NHEJ outcompetes HDR in the race to stably resolve the DSB generated by CRISPR.

A plausible strategy to improve the odds of HDR is to modify the balance between NHEJ and HDR pathways, favoring HDR by either inhibiting NHEJ or activating HDR. Several strategies have been tested to favor HDR by inhibiting canonical NHEJ based on genetic, transcriptional or pharmacological approaches (recently reviewed by Yeh, Richardson, & Corn, 2019). Successful strategies for improving HDR rates have included exposure to chemical inhibitors of key enzymes for the canonical NHEJ pathway such as the ligase IV inhibitor SCR7 or the DNA‐PK inhibitors NU7026, NU7441, or KU‐0060648 (Maruyama et al., 2015; Robert, Barbeau, Ethier, Dostie, & Pelletier, 2015; Singh et al., 2015; Suzuki et al., 2016). Unfortunately, the effects of these inhibitors have been shown to be cell‐type dependent, which has led to the development of complex combinations that are still inefficient in some cell types (Riesenberg & Maricic, 2018). This failure may result from activation of MMEJ instead of HDR following canonical NHEJ inhibition (Nussenzweig & Nussenzweig, 2007), as MMEJ, a relatively newly uncovered repair pathway, has been suggested to be a major contributor to the indels generated following genome editing (Allen et al., 2018; Bae, Kweon, Kim, & Kim, 2014; Shen et al., 2018).

Activation of HDR may be a more direct way to increase the odds of targeted KI compared to canonical NHEJ inhibition. Successful strategies to increase targeted KI efficiency by activating HDR have included (a) the expression of i53, an inhibitor of 53BP1, a key regulator of DSB repair favoring NHEJ over HDR (Canny et al., 2018); (b) the expression of a variant of RAD18 that stimulates HDR (Nambiar et al., 2019); and (c) exposure to the HDR activator RS‐1 (Pan et al., 2016; Pinder, Salsman, & Dellaire, 2015; Song et al., 2016). RS‐1 is a chemical compound (3‐(*N*‐benzylsulfamoyl)‐4‐97 bromo‐*N*‐(4‐bromophenyl)benzamide) that enhances HDR by promoting the formation of active presynaptic filaments (Jayathilaka et al., 2008). Given this background, the aim of this study was to test the effect of RS‐1 on targeted KI rates following CRISPR‐mediated HDR in bovine zygotes.

## 2 RESULTS

**Figure 1:**
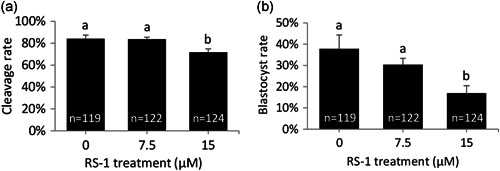
Developmental rates of bovine embryos transiently exposed to 7.5 or 15 µM RS‐1. Cleavage (a) and Day 9 blastocyst (b) rates are depicted. The number of embryos for each group is indicated inside each column. Different letters indicate significant differences based on analysis of variance. *p* < 0.05

A preliminary experiment was conducted to test RS‐1 toxicity in bovine embryos. For this purpose, bovine zygotes were incubated for 24 hr following in vitro fertilization (IVF) in synthetic oviduct fluid (SOF) medium supplemented with RS‐1 at 0, 7.5, or 15 µM and then cultured without RS‐1 for 8 days to determine developmental rates. Embryos that had been transiently exposed to 15 µM RS‐1 exhibited decreased cleavage and blastocyst rates, but transient incubation in 7.5 µM RS‐1 yielded similar developmental rates to those obtained in the control group (Figure 1). On the basis of these results, 7.5 µM was deemed as the highest RS‐1 concentration compatible with normal developmental rates.

**Figure 2:**
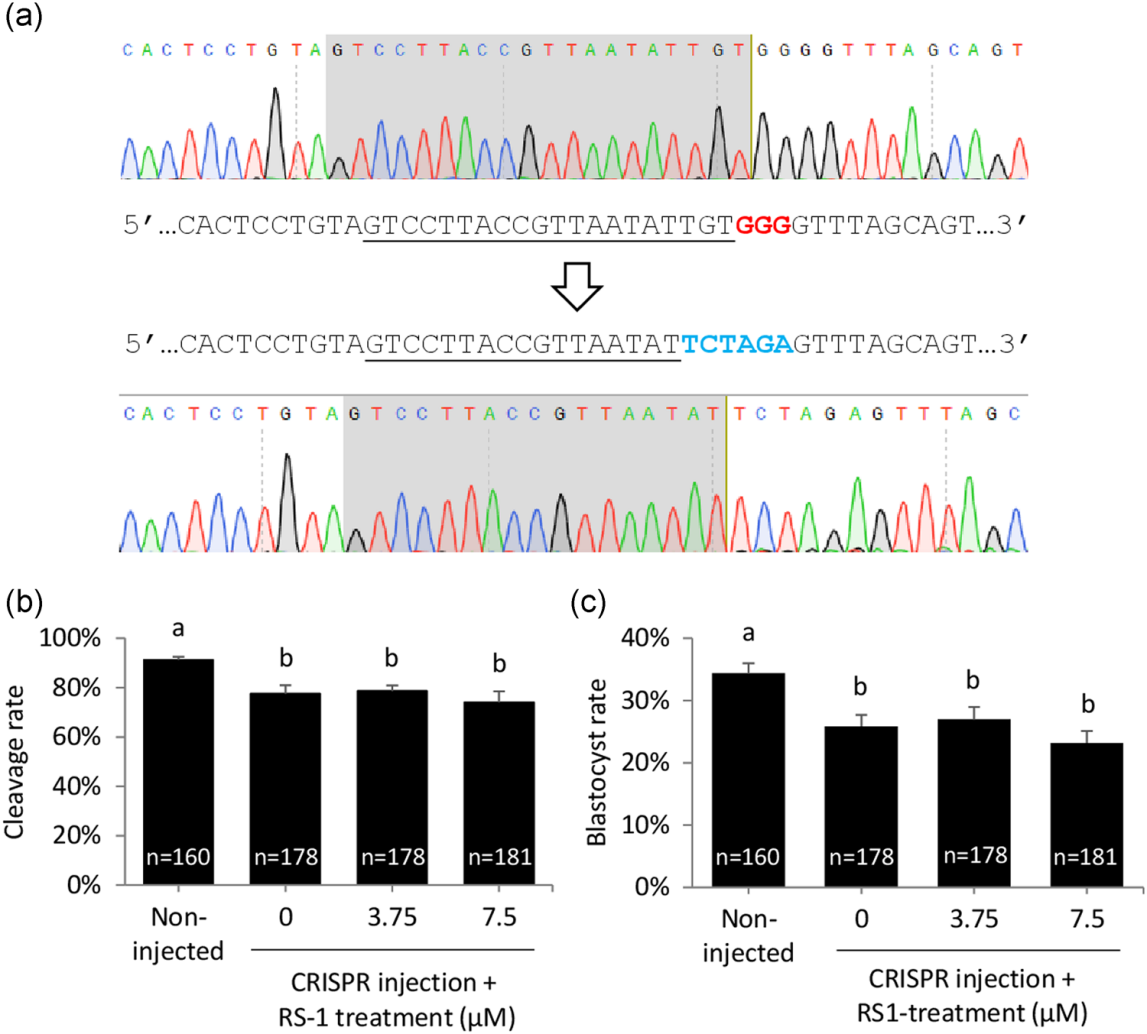
(a) HDR template design. Upper chromatogram corresponds to WT sequence, target sequence is shaded in gray in the chromatogram and underlined in the sequence, PAM (GGG) is marked by red bold letters. Lower chromatogram shows a knocked‐in allele: HDR template was designed to introduce a *Xba*I site (TCTAGA, marked by blue bold letters) substituting six nucleotides including PAM sequence to prevent CRISPR recognition of the edited template. (b,c) Developmental rates of CRISPR‐injected bovine embryos transiently exposed to 0, 3.75 or 7.5 µM RS‐1 compared to non‐injected control. Cleavage (b) and Day 9 blastocyst (c) rates are depicted. The number of embryos for each group is indicated inside each column. Different letters indicate significant differences based on analysis of variance. HDR, homology directed repair; WT, wild type; PAM, protospacer adjacent motif; CRISPR, clustered regularly interspaced short palindromic repeats. *p* < .05

Next, the potential benefit of RS‐1 on CRISPR‐mediated targeted KI was explored. Bovine zygotes were injected with CRISPR components (Cas9 messenger RNA ([RNA], and a single‐stranded DNA [ssDNA] repair template). A noncoding region was used as genomic target to avoid any possible interference of the genomic modification on developmental rates. ssDNA template consisted of an oligonucleotide mediating the insertion of one *Xba*I restriction site flanked by 50 bp homology arms (Table 1). The template was designed to replace six nucleotides of the Wild type sequence including PAM sequence, avoiding CRISPR recognition of the edited template (Figure 2). Following microinjection, presumptive zygotes were randomly allocated into three different groups that were transiently incubated for 24 hr in RS‐1‐free SOF or SOF supplemented with 3.75 or 7.5 µM RS‐1. A group of non‐injected zygotes was kept as control for developmental rates. Cleavage and blastocyst rates were significantly reduced in all injected groups compared to non‐injected embryos but, as expected based on the results of the previous experiment, RS‐1 at a concentration of 3.75 and 7.5 µM did not reduce developmental rates compared to the injected control not exposed to RS‐1 (Figure 2).

**Table 1:** Sequences for genotyping primers and HDR donor. *Xba*I site in HDR donor is underlined

ssDNA	Sequence	Acc. number
Forward primer	CGAACCCTGCCACTACCATT	NC_03738.1
Reverse primer	CCCACCTCCCAACTGCTTAG	NC_03738.1
HDR donor	ACACTGCCCTCTTCCCTTCTCTGCACTCCTGTAGTCCTTACCGTTAATATTCTAGAGTTTAGCAGTCAGTTATATTTCATAGAGTATTTTTCACTAACTCTTACAT	

Abbreviations: HDR, homology directed repair; ssDNA, single‐stranded DNA.

Injected embryos reaching the blastocyst stage were stored and subsequently genotyped to determine genome edition. Genome edition was determined on each blastocyst by Sanger sequencing a polymerase chain reaction (PCR) product containing the target site. This strategy allowed to distinguish between non‐edited and edited embryos (embryos harboring indels generated by NHEJ and/or HDR‐mediated targeted KI). Genome edition rates were unaffected by RS‐1 treatment, being close to 90% in all three groups analyzed (Table 2). To determine targeted KI, the insert (*Xba*I restriction site) was detected by *Xba*I digestion of PCR products followed by gel electrophoresis analysis. A higher rate (*p* < 0.05) of targeted insertion was achieved in embryos exposed to 7.5 µM RS‐1 (53.1%) following microinjection compared with other groups (26.5% and 23.1% for 0 and 3.75 µM, respectively; Table 2). Mosaicism rate was analyzed by clonal sequencing in KI embryos. As expected, given that DNA replication in bovine embryos occurs before the time at which microinjection was done (20 hpi; Lamas‐Toranzo, Galiano‐Cogolludo, et al., 2019), all embryos but one contained more than two alleles. Interestingly, the embryo containing less than two alleles was indeed monoallelic, suggesting that the same KI allele was generated in independent DSB repair events.

**Table 2:** Genome edition, targeted knock‐in (KI) and mosaicism rates

RS‐1 (µM)	Embryos analyzed	Edited embryos (%)	Targeted KI embryos (%)	Mosaic targeted KI embryos (%)
0	45	40 (88.9)	10 (25)^a^	10 (100)
3.75	44	39 (88.6)	9 (23.1)^a^	8 (88.9)
7.5	39	36 (92.3)	19 (52.8)^b^	19 (100)

*Note*: Different superscripts indicate significant differences based on Fisher's exact (*p* < .05).

## 3 DISCUSSION

The generation of targeted KI individuals in livestock species has been achieved by providing an HDR template, alone or combined with CRISPR, to intermediary somatic cells followed by somatic cell nuclear transfer (SCNT; Schnieke et al., 1997; Tan et al., 2013). Given the low efficiency of HDR, this indirect approach eliminates the risk of producing offspring not harboring the intended mutation, as somatic cells containing that mutation can be selected before SCNT. However, the boosted efficiencies of HDR combined with CRISPR allow the direct application of CRISPR + HDR to embryos (Park et al., 2017; Song et al., 2016; Wu et al., 2016), circumventing the need for intermediary cells and the low developmental rates and possible epigenetic defects associated with SCNT (Wilmut, Schnieke, McWhir, Kind, & Campbell, 1997). This latter direct approach requires high KI efficiencies to minimize the number of embryos or animals born without the intended mutation. The high efficiencies of targeted KI achieved using RS‐1 (~50% of the embryos harbored the intended mutation) encourage the use of this direct approach to introduce small insertions.

As targeted KI relies on HDR, several strategies have been developed in cultured cells to control the balance between DSB repair pathways, aiming to increase the chances of HDR. Unfortunately, the success of specific compounds in enhancing targeted KI have proven to be cell‐type specific, and, to date, only two compounds, the NHEJ inhibitor SCR7 and the HDR activator RS‐1, have been tested on mammalian embryos. Conflicting results have been obtained for both compounds in diverse cell types. SCR7 has been found to increase HDR efficiencies (Aslan, Tadjuidje, Zorn, & Cha, 2017; Chu et al., 2015; Hu et al., 2018; Li et al., 2017; Maruyama et al., 2015; Pinder et al., 2015; Robert et al., 2015; Singh et al., 2015), but no significant effects were observed by other groups or on other cell types (Aslan et al., 2017; Canny et al., 2018; Chu et al., 2015; Greco et al., 2016; Gutschner, Haemmerle, Genovese, Draetta, & Chin, 2016; Lee, Grav, Pedersen, Lee, & Kildegaard, 2016; Riesenberg & Maricic, 2018; Song et al., 2016; Xie et al., 2017; D. Yang et al., 2016; Zhang et al., 2017). Similarly, RS‐1 has been reported to both enhance targeted KI (Pan et al., 2016; Pinder et al., 2015; Song et al., 2016) and to not exert a positive effect (Riesenberg & Maricic, 2018; K. Wang et al., 2016; Zhang et al., 2017). Focussing on embryos, SCR7 was observed to increase KI efficiencies in mouse embryos (Maruyama et al., 2015; Singh et al., 2015), but these results could not be replicated in rabbit embryos (Song et al., 2016). To our knowledge, RS‐1 has only been tested in rabbit embryos (Song et al., 2016) where the magnitude of the improvement achieved exceeded that obtained by us in bovine embryos: targeted KI rate increased from 4% to 26% in rabbits, whereas in bovine we found an increase from 25% to 53%. Rabbit embryos were also more tolerant to RS‐1 treatment; a concentration of 15 µM was even reported to enhance blastocyst development compared to the control group (Song et al., 2016), whereas bovine embryo development was reduced when zygotes were transiently exposed to that concentration. In this sense, although the same RS‐1 concentration (7.5 µM) was effective in enhancing targeted KI rates in both species, caution must be taken when extrapolating optimal RS‐1 concentrations from one species to another.

The type of DNA used as template for HDR also impacts targeted KI efficiencies. ssDNA results in higher targeted KI rates than double‐stranded DNA (dsDNA; Ran et al., 2013) and, unlike dsDNA, ssDNA cannot integrate randomly in the genome. Therefore, ssDNA templates are preferable if available, as synthesizing large ssDNA can be difficult. The molecular mechanism by which RS‐1 improved targeted KI using ssDNA as a donor remains unclear. HDR by ssDNA donor occurs by single‐stranded template repair (SSTR), a form of HDR that remains largely unexplored (Yeh et al., 2019). RS‐1 promotes the formation of active presynaptic filaments and stabilizes ssDNA‐RAD51 nucleoprotein filaments, stimulating templated HDR (Jayathilaka et al., 2008). However, SSTR seems to be independent of RAD51, as RAD51 knock‐down reduces HDR efficiencies only when dsDNA donors are used as a template, but not when ssDNA donors are used (Bothmer et al., 2017; Richardson et al., 2018). In this perspective, the mechanism through which RS‐1 enhances HDR using ssDNA as donors (Pan et al., 2016 and our results) may not be mediated by RAD51. Interestingly, at least two RAD51 paralogs, RAD51C and XRCC3, are required for Cas9‐induced SSTR (Richardson et al., 2018), suggesting that SSTR may involve strand invasion in mammalian cells (Yeh et al., 2019) and that RS‐1 may also enhance the activity of these RAD51 paralogs.

Further improvement of targeted KI efficiency may be achieved by other complementary approaches such as modified versions of the CRISPR system that employ Cas9 fused to the repair template (Aird, Lovendahl, St Martin, Harris, & Gordon, 2018; Savic et al., 2018) or to an MRN recruiter (Reuven, Adler, Broennimann, Myers, & Shaul, 2019), or by restricting DSB generation to those phases of the cell cycle when HDR is active: S/G2 (Hustedt & Durocher, 2016). Several approaches have been explored to restrict DSB formation to S/G2 phases such as the use of Cas9 fusion proteins with cell phase‐restricted activity (Charpentier et al., 2018; Gutschner et al., 2016) or cell cycle synchronizers (Zhang et al., 2017). However, an easier approach when CRISPR is applied to embryos is to carefully choose the CRISPR delivery time, as the cell cycle in early embryos is already synchronized by fertilization. By this principle, a significant improvement in HDR rates for large inserts was observed following 2‐cell embryo injection (which display an exceptionally long G2 phase) compared to zygotes (Gu, Posfai, & Rossant, 2018). This improvement is partly due to the duplicated odds for HDR when two cells instead of one are injected, but the improvement achieved (1–7% vs. 32–35%) clearly exceeds the two‐fold increase. In our study, the time of CRISPR microinjection (~20 hr post‐insemination, pi) also coincided with the late S/G2 phase (Lamas‐Toranzo, Galiano‐Cogolludo, et al., 2019), the optimal phase when HDR is intended. Similarly, the previous report employing RS‐1 in rabbit embryos (Song et al., 2016) delivered CRISPR components at 19‐21 hpi, coinciding with the S/G2 phase (Lamas‐Toranzo, Fonseca Balvis, et al., 2019; Oprescu & Thibault, 1965).

In conclusion, exposure to RS‐1 is a successful strategy to enhance HDR, otherwise constrained by competition from NHEJ, and thereby to improve targeted KI rates in bovine zygotes. The two‐fold increase in targeted KI generation rates allows the generation of KI individuals by direct application of CRISPR components to embryos, an easier and faster approach than SCNT‐mediated targeted KI generation.

## 4 MATERIALS AND METHODS

### 4.1 In vitro production of bovine embryos

Bovine ovaries were collected at a local slaughterhouse and transported to the laboratory within 3 hr. Cumulus‐oocyte complexes (COCs) were obtained by aspiration of 2–8 mm diameter follicles and selected based on conventional morphological criteria (Hawk & Wall, 1994). In vitro maturation (IVM) of the selected COCs was performed in TCM‐199 supplemented with 10% (vol/vol) fetal calf serum (FCS) and 10 ng/ml epidermal growth factor at 39°C and 5% CO_2_ in the air with humidified atmosphere for 24 hr. IVF was carried out with frozen‐thawed spermatozoa from a single stud bull selected through a 95% to 45% discontinuous Percoll gradient (Pharmacia). Spermatozoa were diluted to a final concentration of 10^6^ spermatozoa/ml and were co‐incubated with mature oocytes in TALP medium at 39°C and 5% CO_2_ in the air with maximum humidity for 20 hr. Following IVF, cumulus cells were removed by vortexing in phosphate‐buffered saline (PBS) for 3 min. For all experiments, noninjected or CRISPR‐injected zygotes were subsequently cultured in 500 µl of SOF media supplemented with 5% FCS and RS‐1 (Sigma) at different concentrations depending on the group (0, 3.75, 7.5, or 15 µM) for 24 hr under an atmosphere of 5% CO_2_ and 5% O_2_ in air with maximum humidity. After 24 hr, embryos were placed in 25 µl drops of RS‐1‐free culture medium covered with paraffin oil at 39°C, 5% CO_2_, 5% O_2_ in the air with an humidified atmosphere for 8 days. Cleavage and blastocysts rates were assessed at 48 hpi and 9 days post‐insemination and statistical differences were determined by One Way analysis of variance using SigmaStat software. The preliminary toxicity test was performed on three independent replicates, whereas microinjected embryos were obtained from six independent replicates.

### 4.2 Generation and microinjection of CRISPR components

Capped polyadenylated Cas9 messenger RNA (mRNA) was produced by in vitro transcription (mMESSAGE mMACHINE T7 ULTRA kit®; Life Technologies) using as template the plasmid pMJ920 (Addgene 42234) linearized with *Bbs*I and treated with Antarctic phosphatase (NEB). mRNA was purified using MEGAClear kit (Life Technologies). Single guide RNA (sgRNA) was synthesized and purified using Guide‐it sgRNA In Vitro Transcription Kit® (Takara). HDR template consisted of an ssDNA composed of *Xba*I site flanked by 50 bp homology arms. Microinjection was performed immediately after IVF. A solution of 300 ng/µl Cas9 mRNA, 100 ng/µl sgRNA, and 100 ng/µl single‐stranded donor DNA was injected (3‐5 pl) into the cytoplasm using a filament needle under a Nikon Eclipse TE300 microscope. Following microinjection zygotes were randomly and equitably distributed between the three groups of the tested RS‐1 concentrations.

### 4.3 Embryo genotyping

Microinjected embryos were kept in culture until Day 9 post‐insemination. Unhatched blastocysts had their zona removed by brief incubation in acidic PBS (pH 2) to remove any residual spermatozoa and facilitate further digestion. Zona‐free blastocysts were placed at the bottom of a 0.2 ml PCR tube and stored at −80°C. Each blastocyst was digested in 8 µl of Picopure (Thermo Fisher Scientific®) at 65°C for 1 hr followed by inactivation at 95°C for 10 min. For each blastocyst, a genomic sequence including CRISPR target site and spanning beyond donor DNA homology arms was amplified in a 50 µl PCR reaction using 4 µl of the blastocyst digestion product as template. PCR conditions were as follows: 94°C for 2 min; ×40 (94°C for 20 s, 60°C for 30 s, 72°C for 30 s); 72°C for 5 min; hold at 8°C. Forty‐four microliters of the resulting PCR product (368 bp for WT or targeted KI sequence) from each blastocyst were purified using FavorPrep™ PCR Purification Kit (Favorgen). Purified PCR products were Sanger sequenced and analyzed for the presence of genome modifications, generally evidenced by mixed sequencing peaks in their chromatographs. Clean sequence reactions (no mixed peaks around the target site) were aligned with WT sequence, enabling to distinguish between WT embryos and embryos exclusively carrying a single mutated allele. To discern which of the edited embryos harbored the HR‐mediated targeted insertion (*Xba*I site), the remaining 6 µl of PCR products from each blastocyst were incubated with *Xba*I restriction enzyme (NEB) in a digestion volume of 20 µl at 37°C for 2 hr. Digested products were then analyzed through electrophoresis in 2% agarose gels. KI rate was calculated as the number of embryos gaining an *Xba*I restriction site out of the number of edited embryos. Mosaicism rates were determined on KI embryos by clonal sequencing, following the procedure described in (Lamas‐Toranzo, Galiano‐Cogolludo, et al., 2019). Briefly, PCR products were cloned into pMD20 T‐vector (Takara), transformed into competent cells and 10 plasmids/embryo were Sanger sequenced. Statistical differences between groups in edition and KI were assessed by Fisher's exact test using SigmaStat software.

## CONFLICT OF INTERESTS

The authors declare that there are no conflict of interests.
